# Factors Associated with False Positive Breast Cancer Results in the Real-Time Sonoelastography Evaluation of Solid Breast Lesions

**DOI:** 10.3390/medicina60071023

**Published:** 2024-06-21

**Authors:** Ivana Eremici, Andreea Borlea, Catalin Dumitru, Dana Stoian

**Affiliations:** 1 PhD School, Victor Babes University of Medicine and Pharmacy, 300041 Timisoara, Romania; 2 Department of Internal Medicine II, Victor Babes University of Medicine and Pharmacy, 300041 Timisoara, Romania; 3 Obstetrics and Gynecology Department, Victor Babes University of Medicine and Pharmacy, 300041 Timisoara, Romania

**Keywords:** breast cancer screening, real-time sonoelastography, false positive, women

## Abstract

*Background and Objectives*: Breast cancer is one of the most widespread cancers among the female population around the world and is curable if diagnosed in an early stage. Consequently, breast cancer screening imaging techniques have greatly evolved and adjusted over the last decades. Alongside mammography, sonoelastography became an important tool for breast cancer detection. However, sonoelastography still has its limitations, namely, there is still a high occurrence of false positive results in the BIRADS 4 category. The aim of our study is to identify potential false positive predictors and to ascertain the factors influencing the quality of strain ultrasound elastography for the evaluation of suspicious solid breast lesions categorized as BIRADS 4B, 4C, and 5. *Materials and Methods*: We conducted a retrospective study in a single private medical center in Timisoara between January 2017 and January 2022 analyzing 1625 solid breast lesions by the sonoelastography strain using a standardized BIRADS-US lexicon. *Results*: Our study showed that most sonoelastography factors linked to incorrect and overdiagnosis were due to a nodule dimension (OR = 1.02 per unit increase), posterior acoustic shadowing (OR = 12.26), reactive adenopathy (OR = 6.35), and an increased TES score (TES3 OR = 6.60; TES4 OR = 23.02; TES5 OR = 108.24). Regarding patient characteristics, age (OR = 1.09 per unit increase), BMI, (OR = 1.09 per unit increase), and breastfeeding history (OR = 3.00) were observed to increase the likelihood of false positive results. On the other hand, the nodules less likely to be part of the false positive group exhibited the following characteristics: a regular shape (OR = 0.27), homogenous consistency (OR = 0.42), and avascularity (OR = 0.22). *Conclusions*: Older age, high BMI, patients with a breastfeeding history, and those who exhibit the following specific nodule characteristics were most often linked to false positive results: large tumors with posterior acoustic shadowing and high elasticity scores, accompanied by reactive adenopathy. On the other hand, homogenous, avascular nodules with regular shapes were less likely to be misdiagnosed.

## 1. Introduction

Breast cancer is a major public health disease worldwide and a leading cause of death among European women [1,2]. The impact of breast cancer on society led to a continuous expansion and adjustment of breast cancer imaging techniques [3,4]. Among these, mammography has been the only screening method for many years, and its quality has improved by transitioning from film to digital mammography [5]. Nowadays, it is the main tool for breast cancer screening. However, mammography still has drawbacks. It is limited with regards to dense breasts, it lacks precise localization, very small tumors are harder to detect, and it is not suitable for patients younger than 40 [5,6,7,8,9].

These limitations have led to the development and implementation of alternative methods, namely, ultrasound elastography and Magnetic Resonance Imaging (MRI), which are routinely used to evaluate indeterminate and difficult-to-identify breast lesions following mammography [5,10,11].

Sonoelastography is a breast cancer imaging technique that has gained ground lately and is performed routinely as an initial breast cancer detection tool, especially for young patients exhibiting palpable breast lumps or other concerning symptoms [12,13,14,15].

The main advantages of sonoelastography are as follows:

- Painless;
- Non-irradiating;
- Cost-effective;
- Can be performed at any age, including during pregnancy or breastfeeding [16];
- Requires a short examination time;
- It is displayed in real time, and an immediate interpretation can be available;
- It can be used complementarily to mammography in the case of dense breasts;
- It can improve differential diagnosis [4,17,18,19];
- It can detect multicentric or multifocal lesions;
- It can detect the presence of axillary lymphadenopathy [20];
- Last but not least, it can be used as an aid in performing a guided biopsy [21,22].

Breast lesions visualized by ultrasound are reported according to the American College of Radiology Breast Imaging and Data System (ACR BI-RADS) lexicon, which uses standardized terminology based on well-established ultrasound criteria for breast lesion characterization and potential malignancy (BIRADS 1 to 5, increasing with the likelihood of malignancy) [23].

The current ACR guidelines indicate that breast lesions categorized as BIRADS 5 have a higher than 95% risk of malignancy, whilst lesions categorized as BIRADS 3 and below have a lower than 2% risk of malignancy. Between these two values, however, BIRADS 4 lesions carry a high risk of false positive results [24,25,26]. As there is a further subdivision of the BIRADS 4 category in 4A, 4B, and 4C, according to malignancy risk, the highest number of false positives are identified in the 4A category, where up to 90 of biopsies are negative. [12,27,28]. In spite of the greatly improved characterization of solid breast masses by sonoelastography, there is still a high amount of false positive results in the BIRADS 4 category, increasing psychological stress and economic burdens for patients [29,30]. Misdiagnosis by breast sonoelastography can result from various factors, of which the most likely are ultrasound equipment quality, operator error (sonoelastographic technique, subjective evaluation, and experience), and structural aspects of the breast or lesion (size, depth, location, thickness, malignant lesions necrosis, or calcification in benign lesions) [12,31,32,33,34,35,36,37]. As such, the main challenge is to reliably set the criteria required for patient referral for a biopsy in order to minimize the rate of unnecessary biopsies while at the same time not miss any potential malignancies.

The aim of our study is to identify potential false positive predictors and to ascertain the factors influencing the quality of strain ultrasound elastography for the evaluation of suspicious solid breast lesions.

## 2. Materials and Methods

### 2.1. Data Collection

We conducted a study in a single private medical center in Timisoara between January 2017 and January 2022. The research was designed as a retrospective analysis, including 1432 female patients with 1625 solid nodules detected by ultrasound examination.

The inclusion criterion for our study was the presence of any solid breast lesion categorized Birads 4B, 4C, and 5 detected by ultrasound elastography screening in women of all ages. The most common complaints consisted of nodule presence detected by auto examination, breast pain and tenderness, family history of breast cancer, suspicious mammograms, and previous indication for follow-up and nipple discharge.

The exclusion criteria were normal breast and cystic lesions (BIRADS categories 1 and 2), BIRADS 3 and 4A lesions (referred to follow-up), previous breast surgery for malignant lesions, radiation therapy, and prosthetic breast implants.

The gold standard for patients who underwent a biopsy or surgery was represented by a histopathological result.

For each solid lesion, in the same session, a complete ultrasound evaluation consisting of conventional grayscale ultrasound, color Doppler scanning, and strain elastography was performed by an experienced operator (DS).

Alongside the ultrasound morphologic features and elastography characteristics, the following additional information was collected: the affected breast (left or right), the nodule position in the breast using the clockwise lobar approach, age, Body Mass Index (BMI), number of births, and breastfeeding history.

### 2.2. Conventional Ultrasound Evaluation of Solid Breast Lesions

All the patients were evaluated with a HITACHI PREIRUS machine (Hitachi Medical Corporation, Tokyo, Japan), including color Doppler and elastography software (Hitachi Realtime Tissue Elastography “HI-RTE”—Hitachi Medical Systems, Tokyo, Japan). A breast probe, EUP-L53L, 920 mm wide, adapting a dedicated water bag device allowing improved analysis of the superficial layers (skin, fascia, and ligaments), was used to obtain high-resolution conventional B mode images. The patient was in the supine position with both hands raised above the head to fully expose the breasts and axillae. A complete bilateral breast conventional grayscale evaluation was initially performed, following the lobar approach to breast ultrasound instructions, using a ductoradial ultrasonic scanning technique. The probe was held in a horizontal position, perpendicular to the skin, visualizing all of the recommended layers, and starting from the upper layer (skin) to the lower layers (ribs). The evaluation was performed clockwise around the nipple, lobe by lobe. For all lesions, in order to evaluate based on two perpendicular planes, the measurement was performed in a radial and anti-radial orientation [38].

The sonographic evaluation of breast mass features included lesion consistency (only solid lesions), dimension of the lesion (largest diameter), shape, (regular/irregular), margins (well defined/anfractuous/speculated), orientation—the principle axis of the lesion (parallel-“wider than tall”/oblique/not parallel—“taller than wide”), homogeneity (homogenous/inhomogeneous), echo pattern (isoechoic/hyperechoic/hypoechoic/marked hypoechoic), vascularity (no vascularization/perinodal vascularization/intranodal vascularization), calcifications (present or absent), posterior acoustic features (none/enhancement/shadowing), and adenopathy (none/inflammatory/reactive).

The indicative signs of malignancy are represented by irregular shapes, marked hypoechoic, inhomogeneous, spiculated margins, taller than wide, internal vascularization, microcalcifications, and posterior acoustic shadowing accompanied by reactive adenopathy.

Each lesion was described using these features and classified into categories 1 to 5 according to the BI-RADS for breast ultrasound as follows: BIRADS 1, no pathological findings; BIRADS 2, benign; BIRADS 3, probably benign; BIRADS 4, suspicious for malignancy subdivided in 4A (low suspicion), 4B (moderate suspicion), and 4C (high suspicion); and finally, BIRADS 5, highly suggestive of malignancy. BIRADS 1 and 2 lesions were excluded from this study, indicating benign findings. BIRADS 3 and 4A were considered for regrading, and BIRADS 4B, 4C, and 5 were labeled malignant.

### 2.3. Real-Time Elastography Evaluation of Solid Breast Lesions

In addition, real-time strain elastography examination was performed in the same examination session using a 5–18 MHz linear multifrequency probe positioned perpendicular to the skin. Both the qualitative and the semiquantitative techniques were performed. Each lesion was attributed a Tsukuba elasticity score (TES) and a strain ratio (fat-to-lesion ratio—FLR). At least two measurements were performed for each solid nodule.

The Tsukuba elasticity score is graded on a five-point scale based on the visually determined mass stiffness. Based on the color balance observed inside and around the examined tumor, a score of 1 to 5 is attributed to increasing mass stiffness. For qualitative elastography, the following risk categories were considered—low stiffness, TES 1 or 2; intermediate stiffness, TES 3; and high stiffness, TES 4 and 5

For the semiqualitative technique, the strain ratio value was calculated automatically based on determining the average strain measured in a lesion and comparing it to the average strain of a similar area of fatty tissue in the adjacent area. The risk categories for the strain ratio were considered as follows: low stiffness, FLR < 2.8; intermediate stiffness, an FLR between 2.8 and 4.5; and high stiffness, FLR ≥ 4.5.

### 2.4. Final BIRADS Assessment

After performing elastography, the BIRADS score was re-graded as follows. BIRADS scores 3, 4A, and 4B were upgraded if high stiffness was found (ES > 4, FLR > 4.5), BIRADS scores 3 and 4A were downgraded in the case of low stiffness (TES scores of 1 and 2 and FLR < 2.8), and in the case of 4B, 4C, and 5, lesion downgrading was not performed, as per the EFSUMB guidelines.

**Figure 1:**
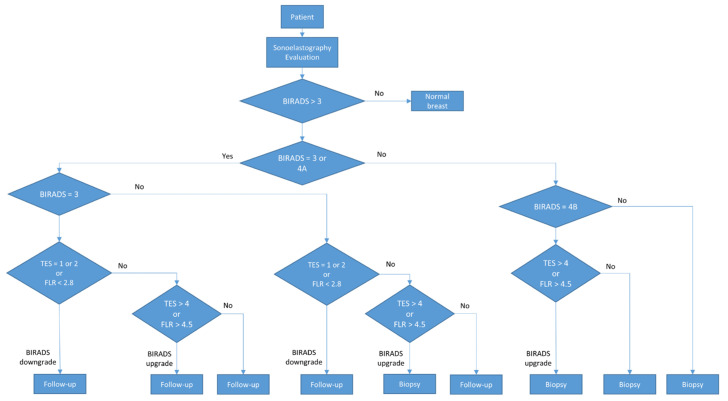
Diagram of the BIRADS score.

During the final assessment, BIRADS categories 3 and 4A were considered low risk and referred to 6–12 months follow-up. BIRADS 4B, 4C, and 5 were considered high risk and referred to a biopsy. The procedure is described in Figure 1.

### 2.5. Statistical Analysis

We conducted a rigorous statistical analysis to provide a comprehensive overview of the study population’s characteristics. Continuous variables were presented based on their distribution characteristics. For normally distributed variables, we reported the mean along with the standard deviation, while for non-normally distributed variables, we opted for the median coupled with the interquartile range. Categorical variables were presented through frequency distributions and proportions. To ensure the reliability of our analysis, we examined the normality assumption of continuous variables using the Shapiro–Wilk test, considering a *p*-value greater than 0.05 as indicative of a Gaussian distribution. Our choice of statistical tests was deliberate and tailored to the nature of the data. We employed the Mann–Whitney U test to explore the differences between continuous variables and utilized the rank biserial coefficient to measure the effect size of these differences. Interpretation of effect sizes followed the established criteria outlined by Funder and Ozer [39], offering nuanced insights into the magnitude of observed differences.

To assess disparities between proportions, we employed Pearson’s chi-squared test, with Cramer’s V coefficient as our measure of effect size. This approach ensured a thorough examination of group differences, with effect sizes interpreted in accordance with established guidelines.

Logistic regression analysis was employed to identify potential risk and protective factors associated with the rate of false positives, incorrect diagnosis, and overdiagnosis. Our variable selection process, guided by the backward elimination method and model optimization based on the AIC (Akaike Information Criterion), ensured the robustness of our findings. To assess the statistical significance of the variables included in the model, we employed the Wald test.

To comprehensively evaluate the performance of our models, we relied on Nagelkerke’s R-squared and ROC (Receiver Operating Characteristics) curve parameters, including accuracy, sensitivity, and specificity, ensuring confidence in our results.

The results were presented employing both graphical and tabular formats. The entire data processing and statistical analysis were conducted using the R programming language version 4.3.0 (R Core Team, Vienna, Austria, 2023). Statistical significance was determined using a significance level of *p*-value < 0.05, with a 95% confidence interval.

## 3. Results

### 3.1. Overview of Variable Summary Statistics in this Study

This study comprised 1625 solid nodules from female patients who presented breast tumors at various stages of development. We collected medical data regarding patient factors (patient age, BMI, history of childbirth and number of children, breastfeeding), as well as characteristics of the breast nodules (size, consistency, homogeneity, echo pattern, shape, margins, orientation, presence and type of nodule vascularization, posterior acoustic features, location, lobar hour orientation, presence of adenopathy, Tsukuba elasticity score, fat-to-lesion ratio). Additionally, medical data related to the histopathological diagnosis (gold standard) and BIRADS (Breast Imaging Reporting and Data System) diagnostic of ultrasound elastography characteristics were collected. A summary of our study characteristics is presented in Table 1 and Table 2.

We observe that all continuous variables included in this study exhibit a non-Gaussian distribution (*p*-value < 0.001), and they are presented as the median and interquartile range. Upon analyzing the study sample, we note that the median age is 41 years, the median BMI is 22.30 years, the nodular size has a median of 9.5 mm, and the FLR index has a median value of 2.32.

### 3.2. Investigating Disparities between the False Positive and Control Groups

We proceeded with the analysis by dividing the study sample into two groups based on the rate of false positive nodules as follows: nodules with BIRADS scores of 4b, 4c, and 5 with a benign histopathological diagnosis were considered false positive nodules, while the remaining nodules were deemed not false positive. Subsequently, we explored the difference between the two groups regarding patient factors and tumor characteristics. The results are presented in Table 3 and Table 4.

We observe that regarding continuous variables, the differences are statistically significant (*p*-value < 0.05). Concerning age, the median age of patients with false positive tumors is higher than those without false positive tumors (52 vs. 40 years), and this difference is statistically significant (*p*-value < 0.001), indicating a very large effect size (−0.48). Regarding BMI, patients with false positive tumors have higher values compared to those without (26.57 vs. 22.27), and this difference is also statistically significant (*p*-value < 0.001) with a large effect size (−0.35). For the size of the nodule, nodules with false positive values exhibit a higher median value (11.20 vs. 9.20 mm), and this difference is statistically significant, with a moderate effect size (*p*-value = 0.004, $\hat{r}$ = −0.21). Additionally, the FLR also presents a higher median value (4.50 vs. 2.29), with the difference being statistically significant and having a very large effect size (*p*-value < 0.001, $\hat{r}$ = −0.46).

Examining the disparities between the two groups regarding the categorical variables in our study, we discern that there are no statistically significant differences in terms of the affected breast, nodule echogenicity, and the clockwise position of the nodule. However, false positive nodules exhibit a higher incidence of patients that performed breastfeeding (83% vs. 48%), and this difference reaches statistical significance (*p*-value < 0.001). Concerning the number of births, patients with false positive nodules display a higher prevalence of individuals with two previous births (65% vs. 21%) and a lower prevalence of those with one (38% vs. 22%) or no births (41% vs. 13%), which is statistically significant (*p*-value < 0.001).

Nodules in the false positive group exhibit a higher occurrence of heterogeneous nodules (49% vs. 22%) compared to the control group, and this difference is statistically significant (*p*-value < 0.001). Likewise, they show a higher prevalence of calcification (19% vs. 4%), significantly different from the control group (*p*-value < 0.001). Regarding nodule shape, false positive nodules exhibit a higher prevalence of irregular shapes (37% vs. 11%), which is a statistically significant difference (*p*-value < 0.001). Additionally, they present a higher prevalence of spiculated and anfractuous margins (16% vs. 5% and 21% vs. 7%, respectively) and a lower prevalence of well-defined margins (89% vs. 63%), which are statistically significant (*p*-value < 0.001). Moreover, false positive nodules show a lower incidence of horizontally oriented nodules (91% s 79%), and this difference is statistically significant (*p*-value < 0.001). They also display a higher prevalence of intranodal (44% vs. 25%) and perinodal (10% vs. 7%) vascularity and a lower prevalence of nodules without vascularity (68% vs. 46%), and those differences are statistically significant (*p*-value < 0.001). False positive cases further demonstrate a higher prevalence of nodules with a posterior acoustic echo pattern (38% vs. 20%), and posterior acoustic shadowing (8% vs. 1%) compared to the control group, and both are statistically significant (*p*-value < 0.001). Lastly, false positive nodules exhibit a higher prevalence of complementary reactive and inflammatory lymph nodes (19% vs. 6% and 17% vs. 12%, respectively) and a lower prevalence of lymph nodes without abnormalities (83% vs. 63%), and these are statistically significant differences (*p*-value < 0.001). They also have a higher prevalence of a TES of 4 (57% vs. 12%), which is a statistically significant difference (*p*-value < 0.001).

**Figure 2:**
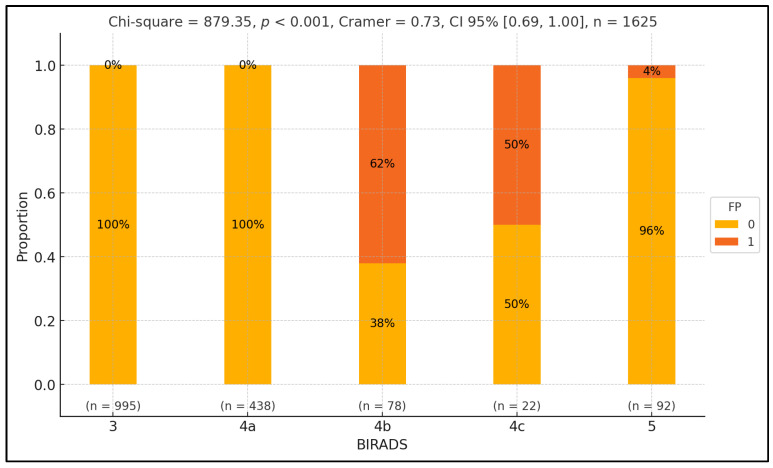
Exploring false positive disparities across BIRADS classes.

To examine variations in false positive rates across different BIRADS classes, we conducted a Pearson chi-squared test. Our analysis revealed a significant discrepancy in false positive proportions across BIRADS classes (*p*-value < 0.001), with a notably substantial effect size ($\hat{V}$ = 0.73). Specifically, we found a higher rate of false positives in the BIRADS 4b class, with the discrepancy reaching statistical significance (*p*-value = 0.04). Conversely, the false positive rate was comparatively lower in the 5 class, and this difference also reached statistical significance (*p*-value < 0.001). Furthermore, BIRADS classes 3 and 4a only consisted of control patients, with statistically significant differences observed across these classes (*p*-value < 0.001), except for BIRADS 4c, in which the proportion between the two groups was equal. The results are summarized in Figure 2.

Figure 2 presents the distribution of false positive (FP) and true positive (TP) rates across different BIRADS classes, represented on the *x*-axis. The *y*-axis shows the percentage of cases within each BIRADS class, ranging from 0% to 100%. Each BIRADS class is illustrated by a vertical bar divided into two segments: green for false positives (1) and orange for true positives (0). Numerical labels inside the bars indicate the proportion of FPT and TP cases. Below each bar, the sample size (n) for each class is noted. At the top, the statistical values include the Pearson chi-squared statistic (879.35, *p* < 0.001), indicating a significant variation in FP rates across BIRADS classes with a substantial effect size (Cramer’s V = 0.73, CI 95% [0.69, 1.00]). The total number of observations is 1625. Individual *p*-values for each BIRADS class are also provided above the bars, highlighting significant differences in FP rates.

### 3.3. Independent Factors That Influence the Rate of False Positives

**Figure 3:**
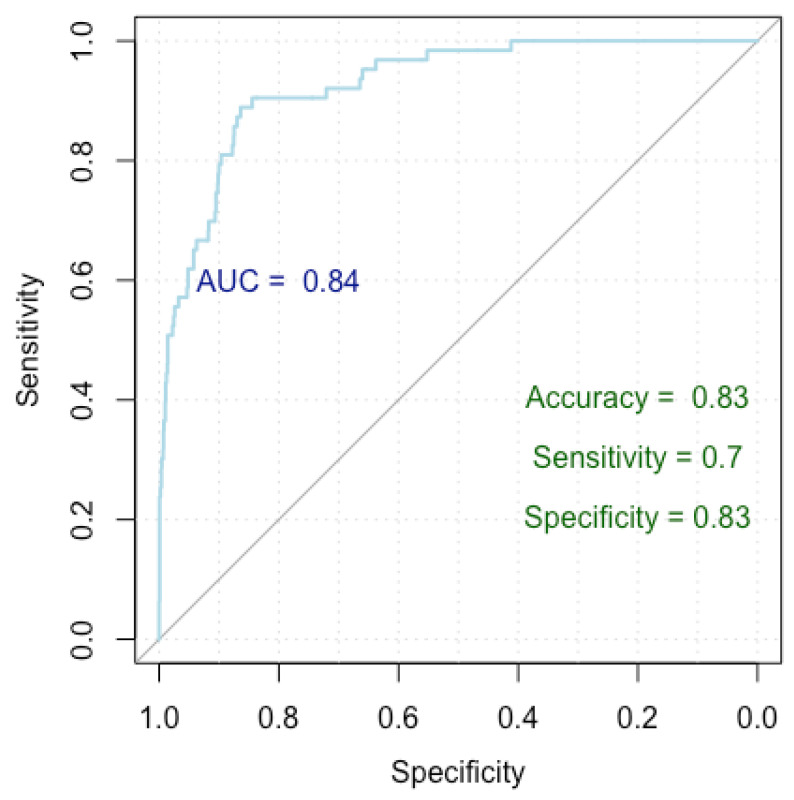
ROC curve for false positive breast cancer identification.

To identify the independent factors influencing the occurrence of false positive nodules, we employed logistic regression analysis. Our findings reveal that nodules characterized by homogeneity and smooth shapes, and those positioned at 4 o’clock and 12 o’clock, exhibit a decreased likelihood of being categorized as false positives. Conversely, nodules with posterior acoustic shadowing are predisposed to a higher likelihood of false positive diagnoses for malignancy. The results are summarized in Table 5 and Figure 3.

Patients experience a 6% increase in the probability of receiving a false positive diagnosis for each additional year of age. This association is statistically significant, with a *p*-value < 0.001 and an odds ratio of 1.06. For every one-year increase in age, there is a 6% higher likelihood of being diagnosed falsely positive.

Patients with a breastfeeding history have a 246% higher probability of receiving a false positive diagnosis compared to those who did not breastfeed. This finding is statistically significant, with a *p*-value < 0.001 and an odds ratio of 3.46.

Homogeneous nodules have a 56% lower probability of receiving a false positive diagnosis compared to the heterogeneous ones, and this result is statistically significant, with a *p*-value of 0.004 and an odds ratio of 0.44.

Nodules with posterior acoustic shadowing are associated with a substantial increase of 1116% in the likelihood of a false positive diagnosis compared to those with no posterior echo pattern. This result is statistically significant, with a *p*-value < 0.001 and an odds ratio of 12.16.

Figure 3 presents a Receiver Operating Characteristic (ROC) curve used to evaluate the performance of our logistic regression model in finding independent risk factors for false positives. The *x*-axis represents specificity (true negative rate) and the *y*-axis represents sensitivity (true positive rate), with the curve plotting the trade-off between these metrics for different threshold values. The Area Under the Curve (AUC) is 0.84, indicating a high level of model discrimination. The model achieved an accuracy of 83%, a sensitivity of 70%, and a specificity of 83%, demonstrating robust performance in predicting false positive diagnoses.

Our model achieved a Nagelkerke’s R-squared of 0.196, explaining 19.6% of the response variable variance. Evaluation of the ROC curve parameters indicates an impressive accuracy of 83%, with a specificity of 83% and a sensitivity of 70%. Furthermore, the AUC index of 0.84 (0.79–0.87 DeLong) underscores the robust performance of our model.

### 3.4. Identifying Independent Factors Linked to an Incorrect Diagnosis

To pinpoint the factors linked to an incorrect diagnosis, we compared false positive nodules to true positive nodules in terms of malignancy diagnosis (confirmed by anatomopathological results considered the gold standard). We utilized logistic regression to delve into the factors that differentiate false positives from true positives. Our analysis reveals notable trends: older age, the presence of reactive adenopathy, and higher TES scores are associated with increased odds of an incorrect diagnosis. Conversely, nodules with regular shapes and absence of nodule vascularization, as well as those with perinodal vascularization, exhibit lower odds of a false diagnosis.

Older age may increase the likelihood of an incorrect diagnosis due to the higher prevalence of benign conditions mimicking malignancy in older populations. Lymphadenopathy can result from infections or inflammation, making benign nodules appear suspicious. Higher TES scores, which are indicative of malignancy, led to more false positives.

In contrast, nodules with a regular shape are typically benign, reducing false positives rates. The absence of nodule vascularization indicates lower malignancy risk, further lowering false positive odds.

**Figure 4:**
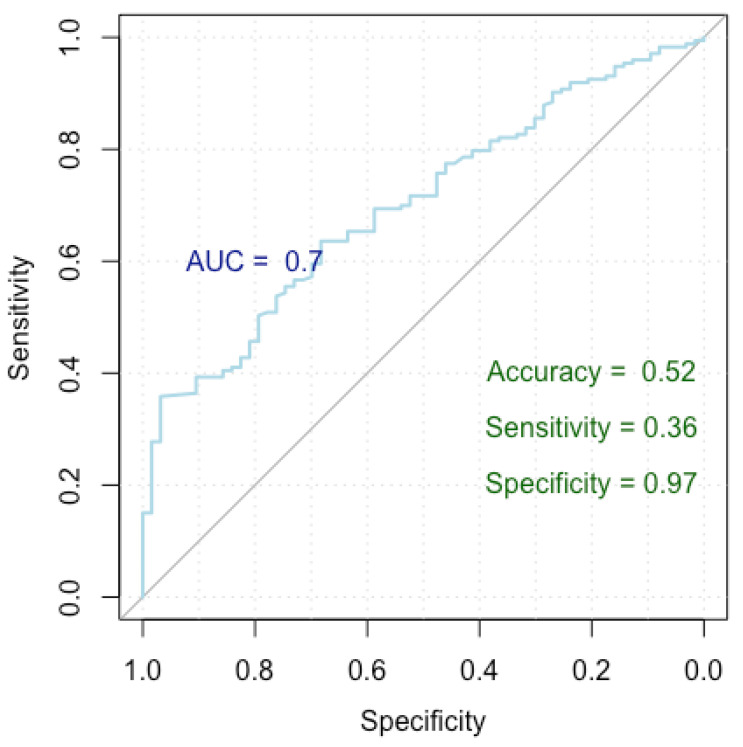
ROC curve for breast cancer incorrect diagnosis.

The results are summarized in Table 6 and Figure 4.

Nodules exhibit diverse probabilities of receiving an incorrect diagnosis based on various factors. With each additional year of age, patients experience a 5% increase in the likelihood of a false diagnosis (OR = 1.05). Nodules with regular shapes demonstrate a roughly 61% lower likelihood of a false diagnosis compared to those with irregular shapes (OD = 0.39). Lack of nodule vascularization is associated with an approximately 78% lower likelihood of a false diagnosis compared to other types (OR = 0.22). Patients with perinodal vascularization show around a 65% lower likelihood of a false diagnosis compared to other vascularization types (OR = 0.35). The presence of reactive adenopathy leads to roughly a 240% higher likelihood of a false diagnosis (OR = 3.40). Nodules with a TES of 3 have approximately a 560% higher likelihood of a false diagnosis compared to those with lower scores (OR = 6.60), those with a TES of 4 have around a 2202% higher likelihood of a false diagnosis (OR = 23.02), and nodules with a TES of 5 have approximately a 10,624% higher likelihood of a false diagnosis (OR = 108.24).

Figure 4 presents a Receiver Operating Characteristic (ROC) curve used to assess the performance of our logistic regression model in identifying factors linked to an incorrect diagnosis, distinguishing between false positive and true positive nodules. The *x*-axis represents specificity (true negative rate) and the *y*-axis represents sensitivity (true positive rate), with the curve showing the trade-off between these metrics at various thresholds. The Area Under the Curve (AUC) is 0.70, indicating moderate discrimination by the model. The model achieved an accuracy of 52%, a sensitivity of 36%, and a specificity of 97%, highlighting its strong ability to correctly identify true negatives, though it has lower sensitivity.

The model achieved a Nagelkerke’s R-squared of 0.605, signifying that it explains 60.5% of the variance in the response variable. In terms of the ROC metrics, the model yielded an AUC of 0.699 (0.630–0.769 DeLong), indicating its strong discriminatory power. Additionally, the model demonstrated an accuracy of 52%, with a specificity and sensitivity of 97% and 36%, respectively.

### 3.5. Unveiling Independent Factors Associated with Overdiagnosis

**Figure 5:**
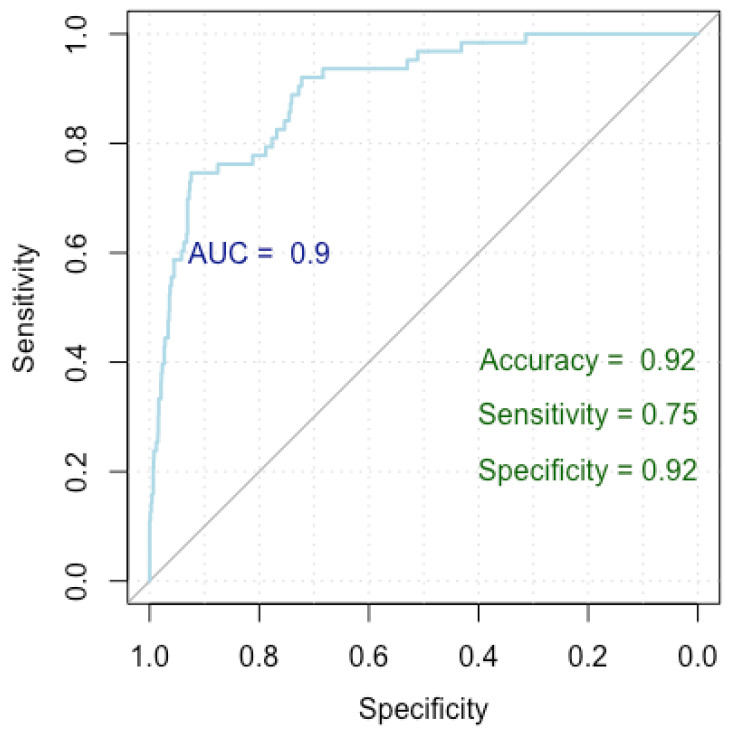
ROC curve for breast cancer overdiagnosis.

To highlight the factors underlying breast cancer overdiagnosis, we developed a logistic regression model to compare false positives cases with true negative cases. Our analysis reveals several significant predictors contributing to the overdiagnosis of breast cancer. Older age, larger nodule dimensions, higher BMI, presence of breastfeeding, presence of nodule calcification, and presence of posterior acoustic shadowing are associated with increased odds of breast cancer overdiagnosis. Conversely, nodules with regular shapes and absence of adenopathy demonstrate lower odds of breast cancer overdiagnosis. Additionally, nodules characterized by homogeneous textures exhibit decreased odds of overdiagnosis compared to heterogeneous nodules. The detailed results are presented in Table 7 and Figure 5.

With each one-year increase in age, there is an approximate 9% rise in the odds of breast cancer overdiagnosis (OR = 1.09). For each unit increase in nodule dimensions, there is a 2% increase in the odds of breast cancer overdiagnosis (OR = 1.02). With each unit increase in BMI, there is a 9% increase in the odds of breast cancer overdiagnosis (OR = 1.09). Patients who breastfed have approximately 200% higher odds of breast cancer overdiagnosis compared to those who did not breastfeed (OR = 3.00). Homogeneous nodules exhibit roughly 58% lower odds of breast cancer overdiagnosis compared to heterogeneous nodules (OR = 0.42). Also, nodules with regular shapes exhibit roughly 73% lower odds of breast overdiagnosis compared to those with irregular shapes (OR = 0.27). Nodules with posterior acoustic shadowing face approximately 1126% higher odds of breast cancer overdiagnosis compared to those without (OR = 12.26). Nodules without adenopathy demonstrate 66% lower odds of breast cancer overdiagnosis compared to those with adenopathy (OR = 0.34), and the ones with reactive adenopathy have approximately 535% higher odds of breast cancer overdiagnosis compared to those with inflammatory adenopathy (OR = 6.35).

Figure 5 presents a Receiver Operating Characteristic (ROC) curve used to evaluate the performance of our logistic regression model in identifying factors associated with breast cancer overdiagnosis, comparing false positive cases to true negative cases. The *x*-axis represents specificity (true negative rate) and the *y*-axis represents sensitivity (true positive rate), with the curve showing the trade-off between these metrics at various thresholds. The Area Under the Curve (AUC) is 0.90, indicating excellent model discrimination. The model achieved an accuracy of 92%, a sensitivity of 75%, and a specificity of 92%, indicating strong performance in identifying true negatives while maintaining good sensitivity.

The model attained a Nagelkerke’s R-squared value of 0.452, elucidating 45.2% of the variance in the response variable. The ROC metrics underscored robust discriminatory power, with an AUC index of 0.927 (0.897–0.957). Additionally, the model achieved an accuracy rate of 92%, with a specificity of 92% and a sensitivity of 75%.

## 4. Discussion

The diagnostic performance of breast cancer screening by sonoelastography is well established, and malignancy criteria of breast lesions are defined and standardized by the ACR guidelines [23]. According to these recommendations, patients exhibiting nodules that are considered low risk (BIRADS 3) are to be referred for a short-term follow-up [27,40]. Starting with BI-RADS 4 and up, the lesions are considered to carry a risk of malignancy and, as a consequence, most biopsied lesions are part of the BIRADS 4 class. Several studies have shown that 69 to 95% of these biopsies are negative [41,42]. The current recommendation is that only nodules classified as 4B and up are to be biopsied, while nodules 4A should be only considered for follow-up due to the high likelihood (>90%) of the subsequent biopsy returning a negative result [13,40,43].

The aim of our study was to highlight the most important factors that lead to false positive results in order to improve the selection criteria for a biopsy.

Although malignancy characteristics are well established, there are still benign lesions falsely identified as potentially malignant, such as complex cystic masses, papillomas, some fibroadenomas, radial scars, fat tissue necrosis, or phyllodes tumors.

Our results are in alignment with the ACR guidelines, as seen in the true positive results for nodules categorized as BIRADS 4B (38%), 4C (50%), and 5 (96%). The expected risk of malignancy as published by the ACR guidelines states malignancy risk values of 2 to 10% for BIRADS 4A, 10 to 50% for BIRADS 4B, and 50 to 95% for BIRADS 4C. In the case of nodules classified as BIRADS 5, the likelihood of malignancy is stated as 95% or greater [23,40].

Regarding the nodule characteristics, we found that nodules in the false positive group compared to the control group exhibited a higher occurrence of heterogeneous nodules (49% vs. 22%) (*p*-value < 0.001), a larger size (11.20 vs. 9.20 mm) (*p*-value = 0.004, =−0.21), calcification (19% vs. 4%) (*p*-value < 0.001), irregular shapes (37% vs. 11%) (*p*-value < 0.001), spiculated and anfractuous margins (16% vs. 5% and 21% vs. 7%, respectively), and a higher prevalence of intranodal (44% vs. 25%) vascularity and posterior acoustic shadowing (8% vs. 1%) (*p*-value < 0.001). The results are in accordance with the existing literature, where a higher number of false positive results were seen in the case of nodules exhibiting microcalcifications [44,45,46,47], posterior acoustic shadowing [32,44], larger nodule dimensions [44], high degrees of vascularity [44,45], and anfractuous or spiculated margins [32].

In our study, the nodules less likely to be part of the false positive group exhibited the following characteristics: regular shapes, homogenous consistency, and avascularity. Homogeneous nodules exhibit roughly 58% lower odds of breast cancer overdiagnosis compared to heterogeneous nodules (OR = 0.42). Nodules with a regular shape exhibit roughly 73% lower odds of breast overdiagnosis compared to those with an irregular shape (OR = 0.27). Lack of nodule vascularization is associated with approximately a 78% lower likelihood of a false diagnosis compared to other types (OR = 0.22).

These results are in accordance with other published work, where breast lesions classified as BIRADS 4 were biopsied and turned out to be benign. Common characteristics of these lesions were regular oval shapes, parallel orientation, and a lack of vascularization. [48,49,50,51,52,53].

A high-stiffness elastogram is another important factor leading to false positives. In our study, the FLR presents a higher median value (4.50 vs. 2.29) for the false positive group when compared to the control group. Regarding the qualitative elastography score, nodules with a TES = 3 have approximately a 560% higher likelihood of a false diagnosis when compared to those with lower scores (OR = 6.60). In the case of nodules given a TES of 4, the likelihood of false positives is around 2202% higher (OR = 23.02). Lastly, nodules with a TES of 5 have approximately a 10624% higher likelihood of a false diagnosis (OR = 108.24). These results are in accordance with the literature regarding the link between increased lesion stiffness and the risk of a false positive diagnosis [32,45,54].

Currently, the examination of axillary lymph nodes is routinely included as part of breast examination, leading to an increase in the incidental detection of abnormal lymph nodes [55,56]. In general, it is well known that the presence of axillary lymphadenopathy is most frequently linked to malignant diseases, such as metastases (mainly from primary breast cancer). In spite of this fact, we have observed in our study that the presence of reactive adenopathy presented increased odds of an incorrect diagnosis by sonoelastography. Nodules without adenopathy demonstrate 66% lower odds of breast cancer overdiagnosis compared to those with adenopathy (OR = 0.34), and the ones with reactive adenopathy have approximately 535% higher odds of breast cancer overdiagnosis compared to those with inflammatory adenopathy (OR = 6.35). This finding agrees with the published literature, where suspicious axillary lymph nodes are not always correlated with the presence of breast cancer. Of these, we can mention other malignancies, infections, and immunological diseases [56,57]. Despite these findings, patients exhibiting axillary lymphadenopathy must be carefully evaluated. General guidance in the case of suspicious axillary lymph nodes is given by the ACR BI-RADS Atlas [23], where increased attention is to be given, especially to enlarged lymph nodes that are new or considerably larger or rounder following previous examination. Detailed patient medical history evaluation must be considered for proper differential diagnosis and further management.

Other factors considered as a potential influence on the rate of false positive results are patients’ age, BMI, history of breastfeeding, and previous births. Our analysis shows that older age, higher BMI, and a history or presence of breastfeeding are associated with increased odds of breast cancer overdiagnosis.

Concerning age, the median age of patients with false positive tumors is higher than those without false positive tumors (52 vs. 40 years) (*p*-value < 0.001). This was also observed in another study, where the likelihood of a false diagnosis was shown to be higher as the patients’ age progressed. The cause could be related to the regression of the lobules and stromal fibers of breasts (glandular—fat tissue ratio). [32,58]. Another age-related aspect to consider is that for the population under 40, no breast cancer screening programs by mammography are established. A major advantage of sonoelastography breast evaluation is that it can be performed for very young patients [16]. Although breast cancer incidence is lower for young patients [59], if present, the disease tends to have a more aggressive behavior with a poor prognosis [60,61,62]. Sonoelastography is a valuable tool in this regard as it can be used to detect malignant breast lesions in an incipient state, potentially improving patient prognosis. Another characteristic of young women is that they tend to exhibit dense breasts [63,64], a feature that is compatible with evaluation by sonoelastography.

We observed an increase in false positive rates in the case of patients with higher BMI (26.57 vs. 22.27) (*p*-value < 0.001). Obesity is known to impede the majority of ultrasound applications [65]. A hypothesis could be that obese women are more likely to have large and adipose breasts, limiting ultrasound evaluation [66,67,68]. Large and fatty breasts are more difficult to evaluate by sonoelastography [21] due to issues related to the penetration depth of ultrasound waves, especially for nodules, which are located in the deeper layers [69]. Patient positioning during examination may be another challenge for obese patients, with larger breasts being more difficult to fix in place when examining by sonoelastography [21].

Lastly, a higher incidence of false positives was observed in patients who have a history of breastfeeding (83% vs. 48%) (*p*-value < 0.001). Physiological changes occur during pregnancy and lactation, which add challenges to breast cancer screening and diagnosis. Post-lactation, due to the remodeling of breast tissue, the breast consistency and structure are altered, resulting in a reduction in breast parenchyma and an increase in fat and connective tissue. [70,71,72]

The strong points of this study are its big sample, evaluation by an experienced sonographer using a high-resolution machine, and a study length of 5 years.

Limitations of this study are represented by its retrospective nature, the fact that not all patients who were referred for follow-up have complied, and finally, for the breastfeeding status, we do not have a distinction between patients who were actively breastfeeding at the time of the examination and patients who have a history of breastfeeding but were not currently at lactation.

## 5. Conclusions

In conclusion, older age, high BMI patients with a breastfeeding history, and those who exhibit the following specific nodule characteristics were most often linked to false positive results: large tumors with calcifications, posterior acoustic shadowing, and high elasticity scores accompanied by reactive adenopathy. On the other hand, homogenous, avascular nodules with regular shapes were less likely to be misdiagnosed.

Taking into consideration that it is nearly impossible to develop an imaging method that has no risk of false positive results in the case of suspect lesions, we need to employ all adequate imaging techniques in addition to clinical examination prior to biopsy referral. Due to the debilitating consequences of missing a malignant breast lesion, a minimally invasive biopsy is preferable in the case of suspect nodules.

## Figures and Tables

**Table 1:** Summary statistics of continuous variables.

Variable	Median	Q25–Q75	*p*-Value
Age	41	34–49	<0.001
BMI	22.30	20.17–25.39	<0.001
Nodule Dimensions (mm)	9.5	6.5–14.2	<0.001
FLR	2.32	1.42–4.00	<0.001

Abbreviations: FLR—fat-to-lesion ratio; BMI—Body Mass Index; Q25–Q75—interquartile range; *p*-value—Shapiro–Wilk test.

**Table 2:** Summary statistics of categorical variables.

Variable	Category	Frequency	Proportion
Births	0	647	39.82%
	1	611	37.60%
	2	367	22.58%
Breastfeeding	Yes	804	49.48%
	No	821	50.52%
Nodule homogeneity	Homogeneous	1254	77.17%
	Heterogeneous	371	22.83%
Nodule calcification	Present	72	4.43%
	Absent	1553	95.57%
Nodule echo pattern	Isoechoic	4	0.25%
	Hyperechoic	5	0.31%
	Hypoechoic	1290	79.38%
	Marked hypoechoic	326	20.06%
Nodule shape	Regular	1429	87.94%
	Irregular	196	12.06%
Nodule margins	Well-defined	1424	87.63%
	Anfractuous	119	7.32%
	Spiculated	82	5.05%
Nodule orientation	Oblique (not parallel)	41	2.52%
	Horizontal (parallel-“wider than tall”	1470	90.46%
	Vertical (not parallel-“taller than wide)”	114	7.02%
Nodule vascularization	No vascularization	1098	67.57%
	Intranodal	415	25.54%
	Perinodal	112	6.89%
Posterior acoustic features	No	1270	78.15%
	Enhancement	332	20.43%
	Shadowing	23	1.42%
Adenopathy	No	1332	81.97%
	Inflammatory	194	11.94%
	Reactive	99	6.09%
Affected breast	Left breast	798	49.11%
	Right breast	827	50.89%
Clockwise position of the nodule	1	182	11.20%
	2	252	15.51%
	3	176	10.83%
	4	126	7.75%
	5	58	3.57%
	6	60	3.69%
	7	100	6.15%
	8	51	3.14%
	9	119	7.32%
	10	193	11.88%
	11	102	6.28%
	12	206	12.68%
TES	1	701	43.14%
	2	360	22.15%
	3	332	20.43%
	4	218	13.42%
	5	92	0.86%
BIRADS	3	995	61.23%
	4a	438	26.95%
	4b	78	4.80%
	4c	22	1.35%
	5	92	5.66%
GS	Benign	1452	89.35%
	Malignant	173	10.65%

TES—Tsukuba elasticity score; BIRADS—Breast Imaging Reporting and Data System; GS—gold standard (histopathological diagnosis).

**Table 3:** Differences between groups regarding continuous variables.

Variable	*p*-Value	$\hat{r}$
Age	<0.001	−0.48
BMI	<0.001	−0.35
Nodule dimension (mm)	0.004	−0.21
FLR	<0.001	−0.46

Abbreviations: FLR—fat-to-lesion ratio; BMI—Body Mass Index; *p*-value—Mann–Whitney U test; $\hat{r}$—rank biserial correlation coefficient.

**Table 4:** Differences between groups regarding categorical variables.

Variable	*p*-Value	$\hat{V}$
Births	<0.001	0.20
Breastfeeding	<0.001	0.13
Nodule homogeneity	<0.001	0.12
Nodule calcification	<0.001	0.14
Nodule echo pattern	0.06	0.05
Nodule shape	<0.001	0.15
Nodule margins	<0.001	0.14
Nodule orientation	0.004	0.08
Nodule vascularization	<0.001	0.09
Posterior acoustic features	<0.001	0.14
Adenopathy	<0.001	0.11
Affected breast	0.19	0.02
Nodule clockwise position	0.47	0.00
TES	<0.001	0.26

TES—Tsukuba elasticity score; $\hat{V}$—Cramer’s V correlation coefficient.

**Table 5:** Independent factors that influence the rate of a false positive diagnosis of malignancy.

Predictors	Odds Ratios	CI	*p*-Value
Age	1.06	1.04–1.08	<0.001
Breastfeeding [yes]	3.46	1.82–7.15	<0.001
Nodule homogeneity [homogeneous]	0.44	0.25–0.78	0.004
Posterior acoustic features [shadowing]	12.16	3.51–36.86	<0.001

Abbreviations: CI—95% confidence interval; *p*-value—the result of the Wald test.

**Table 6:** Factors linked to an incorrect diagnosis.

Predictors	Odds Ratios	CI	*p*-Value
Age	1.05	1.03–1.07	<0.001
Nodule shape [regular]	0.39	0.23–0.66	<0.001
Nodule vascularization [no]	0.22	0.13–0.37	<0.001
Nodule vascularization [perinodal]	0.35	0.14–0.81	0.02
Adenopathy [reactive]	3.40	1.52–7.79	0.003
TES [3]	6.60	2.66–19.98	<0.001
TES [4]	23.02	9.34–69.66	<0.001
TES [5]	108.24	13.82–2400.55	<0.001

Abbreviations: CI—95% confidence interval; *p*-value—the result of the Wald test; TES—Tsukuba elasticity score.

**Table 7:** Factors underlying breast cancer overdiagnosis.

Predictors	Odds Ratios	CI	*p*-Value
Age	1.09	1.06–1.12	<0.001
Nodule dimensions	1.02	1.00–1.05	0.04
BMI	1.09	1.01–1.16	0.024
Breastfeeding [yes]	3.00	1.43–6.85	0.006
Nodule homogeneity [homogeneous]	0.42	0.22–0.84	0.013
Nodule calcification [present]	8.31	3.01–22.86	<0.001
Nodule shape [regular]	0.27	0.13–0.57	0.001
Posterior acoustic features [shadowing]	12.26	3.09–43.98	<0.001
Adenopathy [no]	0.34	0.15–0.82	0.012
Adenopathy [reactive]	6.35	1.97–20.75	0.002

Abbreviations: BMI—Body Mass Index; CI—95% confidence interval; *p*-value—the result of the Wald test.

## Data Availability

The data that support the findings of this study are available upon reasonable request from the corresponding authors. The data are not publicly available due to privacy or ethical restrictions.
